# DEMO-II Trial. Aerobic Exercise versus Stretching Exercise in Patients with Major Depression—A Randomised Clinical Trial

**DOI:** 10.1371/journal.pone.0048316

**Published:** 2012-10-31

**Authors:** Jesper Krogh, Poul Videbech, Carsten Thomsen, Christian Gluud, Merete Nordentoft

**Affiliations:** 1 Mental Health Centre Copenhagen, Faculty of Health Sciences, University of Copenhagen, Copenhagen, Denmark; 2 Institute for Basic Psychiatric Research, Department of Biological Psychiatry, Psychiatric Hospital, Århus, Risskov, Denmark; 3 Department of Radiology, Rigshospitalet, Faculty of Health Sciences, University of Copenhagen, Copenhagen, Denmark; 4 Copenhagen Trial Unit, Center for Clinical Intervention Research, Rigshospitalet, Copenhagen University Hospital, Copenhagen, Denmark; University of Bath, United Kingdom

## Abstract

**Background:**

The effect of referring patients from a clinical setting to a pragmatic exercise intervention for depressive symptoms, cognitive function, and metabolic variables has yet to be determined.

**Methods:**

Outpatients with major depression (DSM-IV) were allocated to supervised aerobic or stretching exercise groups during a three months period. The primary outcome was the Hamilton depression score (HAM-D_17_). Secondary outcomes were cognitive function, cardiovascular risk markers, and employment related outcomes.

**Results:**

56 participants were allocated to the aerobic exercise intervention versus 59 participants to the stretching exercise group. Post intervention the mean difference between groups was −0.78 points on the HAM-D_17_ (95% CI −3.2 to 1.6; *P* = .52). At follow-up, the participants in the aerobic exercise group had higher maximal oxygen uptake (mean difference 4.4 l/kg/min; 95% CI 1.7 to 7.0; *P* = .001) and visuospatial memory on Rey’s Complex Figure Test (mean difference 3.2 points; 95% CI 0.9 to 5.5; *P* = .007) and lower blood glucose levels (mean difference 0.2 mmol/l; 95% CI 0.0 to 0.5; *P* = .04) and waist circumference (mean difference 2.2 cm; 95% CI 0.3 to 4.1; *P* = .02) compared with the stretching exercise group.

**Conclusions:**

The results of this trial does not support any antidepressant effect of referring patients with major depression to a three months aerobic exercise program. Due to lower recruitment than anticipated, the trial was terminated prior to reaching the pre-defined sample size of 212 participants; therefore the results should be interpreted in that context. However, the DEMO-II trial does suggest that an exercise program for patients with depression offer positive short-term effects on maximal oxygen uptake, visuospatial memory, fasting glucose levels, and waist circumference.

**Trial Registration:**

ClinicalTrials.gov NCT00695552

## Introduction

Based on the current development in disease patterns, unipolar depression is expected to be the second highest contributing factor to the global disease burden in 2030 [1]. Depression is characterized as a mood disorder and often associated with impaired cognitive skills [2] and increased mortality from cardiovascular and endocrinological diseases [3]. First-line treatment for moderate depression is usually antidepressant monotherapy, psychotherapy, or a combination of both [4]. Low compliance and high drop-out rates during antidepressant therapy could explain why only 50% are expected to remit during the acute phase of treatment [5]–[7]. This has resulted in the interest and evaluation of various forms of alternative and complementary therapies in depressed patients.

Exercise as an antidepressant has been the focus of several trials [8]. In addition to the potential antidepressant properties, exercise has shown to increase cognitive function in selected populations [9] as well as improving the cardiovascular risk profile [10]. Pre-clinical studies suggests that the exercise induces a central increase in neutrophines [11] and monoamines [12], which offers a potential explanation for exercise as an antidepressant. The authors previously conducted a clinical trial of exercise in depressed patients (DEMO-I) and found no convincing antidepressant effect of allocating patients to either strength training or aerobic exercise compared with an attention control [13]. These non-significant findings could potentially be explained by the low exercise frequency (two days per week) and a heterogeneous population with regard to antidepressant treatment [13]. On the other hand the DEMO-I trial did suggest that exercise could have a positive effect on absence from work in this patient group [13].

A recently conducted systematic review of randomized trials investigating the antidepressant properties related to exercise in clinically depressed patients found a significant effect of exercise (standardized mean difference of −0.40 and 95% confidence interval of −0.7 to −0.1). By pooling trial results with adequately concealed random allocation, blinded outcome assessment, and intention-to-treat analysis, the standardized mean difference was reduced to −0.19 (95% CI –0.7 to 0.3) and no longer significant [8]. In addition, only a few of the included studies were conducted in health care settings where exercise might plausibly be prescribed to patients.

The current randomized trial was designed to address several issues. We hypothesized that a pragmatic aerobic exercise intervention would have antidepressant properties in a group of clinically depressed patient’s. We also hypothesized that aerobic exercise would have a positive effect on cognitive function in depressed patients, which has only been investigated in two trials with contradicting results [13], [14]. Moreover, we expected that exercise would have a positive effect on risk markers of cardiovascular disease and absence from work in this patient group.

## Methods

The protocol for this trial and supporting CONSORT checklist are available as supporting information; see Checklist S1 and Protocol S1.

### Trial Design

This was a single centre, two-armed, parallel-group, observer-blinded randomized clinical superiority trial. The participants were enrolled at the trial site in Copenhagen (Denmark) and randomized to either aerobic exercise or an attention control group (i.e., stretching exercise).

### Procedure

We informed general practitioners and the public about the trial through advertisements in local papers, leaflets, and a website. All participants had to be referred by a physician or a psychologist. Referred participants were invited to an interview, and in case the participant fulfilled all the inclusion criteria and none of the exclusion criteria, they were offered inclusion. Psychological and cognitive assessment took place at this occasion while physical outcomes were assessed within a week. Subsequent to the physical assessment, the participants were randomized to one of two interventions. After three months of intervention, participants were invited to an interview, which assessed all psychological, cognitive and employment related outcomes. At the participants’ convenience the assessment of physical outcomes could take place at another day. The participants were offered 500 DKK (66 EUR) as compensation for participation in the final outcome assessments.

### Trial Staff

Assessment of participants’ eligibility and outcome assessments were undertaken by a trained psychiatric nurse in the majority of patients. Otherwise this was performed by one of the investigators (JK). The nurse had received training in using the outcome assessment tools prior to the first participant and received continuous supervision throughout the trial. The intra class correlation between the two raters was 0.91 (95% CI 0.62 to 0.98) for the primary outcome (HAM-D_17_). Certified physiotherapists conducted the intervention.

### Participants

Eligible participants were men and women between 18 and 60 years of age, referred from a clinical setting by a physician or a psychologist, a diagnose of major depression (DSM-IV) based on the Danish version of the Mini International Neuropsychiatric Interview [15]. The participants all scored above 12 on the HAM-D_17_ and were living in the Greater Copenhagen catchments area. They were able to comprehend and sign the informed consent statement. Exclusion criteria were current drug abuse, any antidepressant medication within the last two months, current psychotherapeutic treatment, contraindications to physical exercise, more than 1 hour of recreational exercise per week, suicidal behaviour according to the 17-item Hamilton depression rating scale (HAM-D_17,_ item 3>2), pregnancy, current/previous psychotic or manic symptoms, or lack of informed consent.

### Randomization

Participants were randomized with a 1∶1 ratio to either aerobic exercise versus an attention control group. Randomization was stratified according to severity of depression (high or low depression score: >17 HAM-D_17_) and blood pressure (high or low blood pressure: >140/95 mmHg). The randomization was centralized and carried out by the Copenhagen Trial Unit (CTU) using a computerized randomization sequence with alternating block sizes (alternating 8, 10, and 12) unknown to the investigators. Prior to the first training session of the participant, the trial physiotherapist would contact the CTU by phone for participant allocation.

### Blinding

Neither participants nor the physiotherapist conducting the intervention were blinded to the allocation. The outcome assessors (the study nurse and the laboratory technicians) were all blinded to participant allocation. Prior to the follow-up interview, participants were instructed not to reveal their allocation to the outcome assessors. The statistical analysis and preparation of the first draft was carried out blinded to group assignments.

### Interventions

Both intervention groups were scheduled to meet three times per week for three months for a total of 36 sessions. All sessions were conducted in the afternoon by a physiotherapist. If the participants did not attend the planned session, the physiotherapists were instructed to contact the participants by phone or by using text messages.

The program of the aerobic training group was designed to increase fitness as measured by maximal oxygen uptake (VO_2max_). After initial 10 minutes of general low-intensity warm-up, the participants did 30 minutes of aerobic exercise on a stationary cycle ergometer (Monark®) followed by five minutes low-intensity cool down period. During the initial four weeks, the aim was to work out at intensity levels corresponding to at least 65% of their maximal capacity, progressing to 70% and 80% during the second and third month, respectively. The participants carried a pulse monitor (Polar m-31®) during exercise sessions to guide and document intensity levels.

The stretching exercise group was designed as an attention control group with the purpose of providing the same level of social interaction and contact with health care professionals as in the aerobic exercise group. This was done in order to assess the potential antidepressant effect of aerobic exercise in it self, and not the effect of aerobic exercise plus social interaction. This stretching exercise group performed low intensity exercise, which we did not expect to contain any antidepressant effect per se. The initial 10 minutes were low-intensity warm-up on a stationary bike, then a 20 minutes program of stretching, followed by 15 minutes of various low intensity exercises such as throwing and catching balls.

### Outcome Measures

#### Psychiatric

The primary outcome measure was the total HAM-D_17_ score measured post-intervention. The HAM-D_17_ is a structured interviewer based questionnaire containing 17 questions rated from 0–2 or 0–4 amounting to a total between 0 and 52 points. A high score reflects a higher level of depressive symptoms. The time frame for evaluation is set to the past two weeks including the day of administration. A score between 12 to 18 points indicates mild depression, 18 to 24 points moderate depression, and more than 24 points severe depression [16]. The retest reliability for this scale has been reported to be between 0.81 and 0.98 [17]. Based on the HAM-D_17_ assessment, we also report core depression items (HAM-D_6_: item 1, 2, 7, 8, 10 and 13) [18], and remission defined as not full-filling the DSM-IV criteria for major depression and a HAM-D_17_ score below 8. The participants filled in the Beck Depression Inventory (BDI) [19] each week as a measure of self reported depression. The BDI-II is a participant administered questionnaire with 21 items. The total score ranges 0 through 63 points. A lower score means less depression. In addition, the WHO-5 well-being scale was used, which is a 5-item participant administered questionnaire with a total score ranging from 0 through 100 points [18]. A high score reflects a higher level of well-being. Anxiety was measured by the Hamilton Anxiety scale (HAM-A_14_) [20]0 trough 56 where a high score reflects a higher anxiety level.

#### Intelligence

We used the Danish Adult Reading [21]Test (DART) [22], which is a Danish version of the New Adult Reading Test [22] comprised of A list of 50 irregularly spelled words. The participant was asked to read out aloud the list of words and the number of correctly pronounced words was used as a proxy-measure of vocabulary intelligence.

#### Memory

The Buschke Selective Reminding Test [23] is a verbal memory test. A list of ten different unrelated words is read aloud to the participant. The participant is then asked to recall the list. The interviewer repeats the words that the patient misses and the participant is asked to try again until all ten words can be said, or until ten attempts. The score is the total number of blanks or mistakes; thus, a high score indicates poorer performance. Visuospatial memory was tested using the Rey’s Complex Figure Test [24]. In this test the participant is shown a geometrically complex figure on a sheet of paper and asked to copy it to another sheet of paper. When this is done the drawings and the original are put away and after three minutes the participant is asked to draw as much of the figure they can recall. The score is calculated based on the three-minute recall drawing. A high score reflects better performance.

#### Attention

Attention was measured using the Digit Span Test [25] whereby the participant repeat orally given strings of digits of increasing lengths in straight and reversed order. The number of correctly repeated strings is the score. Subtracting Serial Sevens [26] requires the participant to subtract seven from 100 and continue to subtract seven until around zero. The score (1–10) is a combination of time and number of errors. The higher score the better performance. The Stroop test [27] included two worksheets read out separately by the participants. Each sheet of paper presents 100 words in different colors. Sheet number one is the congruent condition, where the word with matching color (e.g., the word ‘green’ was printed in green) is to be named. Sheet number two presented the incongruent condition, in which the color of the mismatching words should be named (e.g. the word ‘yellow’ was printed in blue). The participants were asked to name the colors of the words as fast as possible and the time taken to complete the task was recorded as the score.

#### Psychomotor speed

Trail Making A and B [28] were used to assess psychomotor speed. In part A, the participants are asked to connect numbered circles on a sheet in consecutive order. In the B part they are asked to connect numbers and letters in alternating sequence (A-1–B-2–C-3…). The score on each test is the time to complete, the faster the better. The Digit Symbol Test [25] is a symbol/number substitution test where the participants are presented with the numbers 1–9 written at the top of a piece of paper. Each number is represented by a symbol. Below there are 100 symbols listed but without the corresponding number. The participant is asked to fill in the corresponding numbers in 90 seconds. The number of correct matches calculates the score, the higher the better.

#### Verbal fluency

Verbal Fluency S and Animals [29] tested the language: In the S part, the participants are asked to name as many words beginning with the letter S as possible. They are not allowed to use proper nouns. In the Animal part, the participants are asked to mention as many animals that they can think of. In both tests the participants have 60 seconds to name as many as possible. The score in each test corresponds to the number of correct words minus the incorrect, the higher the better.

#### Physical examination

For the physical examination, the participants were requested to meet at the research department between 8∶00 and 10∶00 a.m. The participants were instructed not to take any food or liquids except for water beginning from midnight prior to the examination and abstain from strenuous physical activity prior to the examination. Height and weight was measured using an electronic weight (Sohnle Medical®, Type 7700, Backnang, Germany) Waist circumference was assessed by standardized procedures and reported as the mean of two measurements. Blood pressure was obtained after five minutes rest with the participant in a sitting position using a certified digital blood pressure monitor (Omron M6, Omron Healthcare co. LTD, Kyoto, Japan). The average of three measurements using the right arm is reported. An indwelling venous catheter was inserted in the ante-cubital vein and blood samples collected after 5 minutes rest in a sitting position. Blood samples were immediately sent to the laboratory for analysis by automated procedures (Modular, P- Modul, Roche Diagnostics, Indianapolis, USA) testing total cholesterol, triglycerides, high density lipoproteins (HDL), fasting glucose, insulin and ELISA for high sensitive c reactive protein (hsCRP). The patients’ VO_2 max_ was estimated using a bicycle cardiopulmonary exercise test (Ergomedic 839 e, Monark, Vansbro, Sweden) based on L. B. Andersen’s cycle exercise protocol [30].

#### Employment status

We recorded the employment status of the participants as well as whether they were on sick leave at the time of the interview. For participants in employment, we registered the number of days spent on the job within the last ten working days expressed as a percentage.

### Sample Size Calculation

Based on our previous experience, we estimated the standard deviation of the HAM-D_17_ total score post-intervention to be six points [13]. Based on this standard deviation, a two-sided type 1 error probability of 5%, and a power of 90%, we should include 85 participants in each intervention group to detect a minimal relevant difference of 3 points on the HAM-D_17_ scale. Anticipating a 20% unavailability for follow-up assessment, based on previous research, the present study aimed to enroll 212 participants to offset the potential loss of power.

### Statistics

The statistical analysis was based on the intention-to-treat principle including all randomized patients regardless of subsequent withdrawal or deviation from the protocol. Clinical effectiveness may be overestimated if an intention to treat analysis with multiple imputation is not conducted [31]. Assessment of variables measured at baseline and post-intervention was reported using mean and standard deviation or if appropriate median and the interquartile range (25^th^ and the 75^th^ percentile). All continuous outcome measures were analyzed using a repeated measurement linear mixed-effects model with an unstructured variance matrix. The intervention effect was assessed by the group × time interaction term. The mixed-effects function is able to handle missing continuous data using a likelihood estimation of missing data [32]. Dichotomous outcomes were assessed with odds ratios and chi-square tests. Missing data on dichotomous outcomes were analyzed by multiple imputations using the MI function in SPSS. Per-protocol analysis was undertaken including participants who had attended one or more sessions per week.

The a priori defined primary outcome were considered significant if p≤0.05. For all other outcomes, we considered p≤0.01 as significant findings while adjusting for multiple testing. For these outcomes, p values between 0.01 and 0.05 were designated as trends. All significance tests are two-sided. We analyzed data using SPSS version 19.0 (SPSS, Inc.; Chicago, USA).

### Protocol Deviations

We introduced the HAM-D_6_ as an outcome measure after recruitment had been initiated. Furthermore, we omitted pulse monitoring in the attention control group, since this encouraged participants to increase their workload during the warm-up.

### Approvals

The trial protocol was approved by the local ethics committee (H-A-2008-046), the Danish Data Protection Agency (J.nr.2008-41-2354), and registered at ClinicalTrials.gov (NCT00695552). Verbal and written informed consent was obtained from all participants involved in the trial.

## Results

### Participants

**Figure 1:**
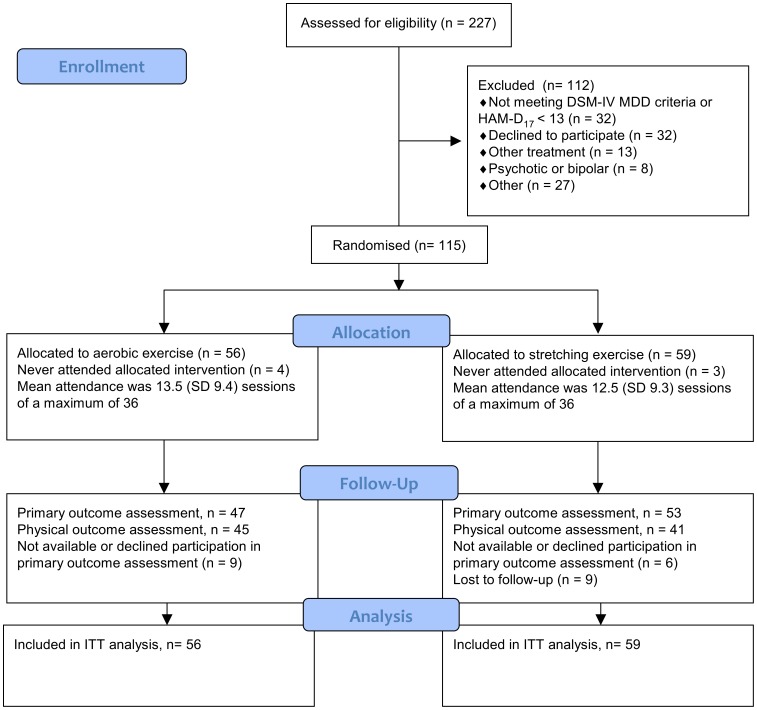
Flow diagram for the DEMO-II trial.

Between September 2008 and April 2011, 227 potential participants were referred to the trial site from various clinical settings. Of these, 112 were excluded and 115 patients were enrolled and randomized; 56 were allocated to aerobic exercise versus 59 to the stretching exercise group. The primary reasons for exclusion were a failure to meet the criteria for depression (n = 32) or declining participation (n = 32). Please see figure 1 for details on participant flow. The majority of participants were referred from general practitioners (111/115) and four participants from private practicing psychiatrists or psychologists. The mean age in the enrolled group was 41.6 years (95% CI 19 to 59) and consisted of 77/115 (67%) female participants. The mean HAM-D_17_ was 18.9 points (95% CI 13 to 28) indicating mild to moderate depression, 59/115 (51.3%) had recurrent depression, and 67/115 (58.3%) had co-morbid generalized anxiety disorder.

The aerobic exercise group and the exercise control group were generally comparable with respect to baseline demographic and clinical characteristics as displayed in table 1. Participants allocated to the aerobic exercise group were slightly younger (mean diff. 3.7 years; 95% CI –0.4 to 7.9), had lower scores at the WHO-5 well-being index (mean diff. 4.7; 95% CI 0.9 to 8.5), and had slightly higher scores at the verbal fluency test (mean diff. 2.3 words; 95% CI 0.1 to 4.5) compared to the stretching exercise group.

### Compliance

The mean attendance was 13.5 (range 0 to 34 and SD 9.4) sessions in the aerobic exercise group versus 12.5 sessions (range 0 to 34 and SD 9.3) in the stretching exercise group (mean difference = −0.98 sessions; 95% CI −4.4 to 2.5) of a planned total of 36 sessions. This corresponds to an average participation of one session per week.

### Follow-up

Post-intervention 47/56 (83.9%) from the aerobic exercise group versus 53/59 (89.8%) from the stretching exercise group attended the psychological assessment interview (chi-square = 0.89; df = 1; p = 0.35). Physical assessment post-intervention was completed by 45/56 (80.4%) allocated to the aerobic exercise group versus 41/59 (69.5%) in the stretching exercise group (chi-square 1.80; df = 1; p = 0.18). These data suggest that the attrition at follow-up assessment was not skewed and further analysis did not reveal significant baseline differences between participants who attended the follow-up interview or physical assessment on sex, age, unemployment, HAM-D_17_, VO_2 max_ or number of previous episodes with depression.

**Table 1:** Characteristics of participants at baseline.

	Aerobic exercise (n = 56)	Stretching exercise (n = 59)
Age, years	39.7 (11.3)	43.4 (11.2)
Female, n (%)	40 (71.4)	37 (62.7)
**Depression**		
HAM-D_17_	19.2 (4.7)	18.6 (4.0)
Total HAM-D_17_>17	36 (64.3%)	38 (64.4%)
HAM-D_6_	10.6 (2.4)	10.2 (2.2)
HAM-A_14_	18.1 (5.2)	17.1 (5.7)
BDI	35.7 (7.0)	35.5 (8.4)
WHO-5	17.8 (8.4)	22.5 (11.7)
Depression, single, n (%)	26 (46.4%)	30 (50.8%)
Depression, recurrent, n (%)	30 (53.6%)	29 (49.2%)
Time since diagnosis, months (IQR)	6.0 (2.6 to 18.0)	6.0 (3.0 to 24.0)
Suicide attempt. <12 months, n (%)	5 (8.9%)	2 (3.4%)
Generalised anxiety, n (%)	33 (58.9%)	34 (55.9%)
**Cognitive function**		
** **Danish Adult Reading Test	32.9 (11.2)	34.7 (7.5)
Buschke’s SRT	16.2 (11.5)	17.7 (5.9)
RCFT –3 min. recall	20.9 (6.3)	20.9 (6.7)
Digit span, forwards	6.5 (2.1)	6.3 (2.2)
Digit span, backwards	5.4 (2.0)	5.0 (1.9)
Subtracting Seriel Sevens	7.6 (2.9)	8.2 (2.5)
Stoop’s test, congruent	60.5 (14.3)	67.4 (56.0)
Stroop’s test, incongruent	123.8 (29.0)	130.5 (43.6)
Trail making A	32.3 (11.0)	35.5 (17.1)
Trail making B	73.5 (23.0)	83.6 (36.1)
Digit symbol test	46.2 (10.1)	45.6 (9.9)
Verbal fluency - animals	22.0 (6.1)	19.7 (5.9)
Verbal fluency – s words	12.0 (4.3)	12.1 (5.4)
**Somatic**		
Weight, kg	76.3 (21.5)	77.4 (17.7)
Body mass index	26.2 (6.2)	26.5 (5.7)
Waist circumference, cm	88.5 (17.9)	90.2 (15.1)
VO_2 max_ ml/kg/min	25.9 (6.9)	26.0 (11.7)
Cholesterol, mmol/l	5.2 (1.1)	5.2 (1.2)
Triglycerides, mmol/l	1.3 (0.8)	1.4 (0.7)
HDL, mmol/l	1.5 (0.3)	1.4 (0.4)
Glucose, mmol/l	5.1 (0.6)	5.1 (0.8)
Insulin, pmol/l	66.7 (51.0)	69.8 (55.5)
QUICKI	0.68 (0.27)	0.94 (2.04)
hsCRP, mg/l	2.4 (3.03)	2.6 (4.3)
Systolic BP, mmHg	121.6 (15.6)	123.0 (18.4)
Diastolic BP, mmHg	81.3 (10.5)	82.4 (12.8)
Hypertension, n (%)	11 (19.6%)	12 (20.3%)
**Work**		
Unemployed, n (%)	20 (35.7%)	27 (45.7%)
Sickness leave, n (%)	20 (35.7%)	18 (30.5%)
Job attendance, last 10 days, %	74.3 (39)	73.8 (34)

Data are presented with mean (SD) unless stated otherwise.

Abbreviations: HAM-D_17_/HAM-D_6_ – Hamilton depression scale with 17 or 6 Items; BDI – Beck’s Depression Inventory II; WHO-5 – World Health Organisation’s well being index; IQR – interquartile range; Buschke’s SRT – Buschke’s Selective Reminding Test; RCFT – Rey’s Complex Figure Test; VO_2 max_ – maximal oxygen uptake; QUICKI – Quantitative insulin sensitivity index; hsCRP – high sensitive C reactive Protein.

### Primary Outcome - Depression

As displayed in table 2 the estimated HAM-D_17_ rating post-intervention in participants allocated to intervention aerobic exercise was 11.3 (SD 6.6) versus 10.5 (SD 6.4) in the stretching exercise group. The estimated mean difference in change from baseline was −0.78 points on the HAM-D_17_ (95% CI. −3.2 to 1.6) in non-significant favor of the stretching exercise group. The participants’ self- assessment of depressive symptoms throughout the trial using the Beck’s Depression Inventory did not suggest any differential effect of interventions (group × time; F_11, 50_ = 1.22; p = 0.30).

**Table 2:** Patients with depression randomly allocated to an aerobic exercise group or a stretching exercise control group in a three months exercise intervention.

	Aerobic exercise(n = 56)	Stretchingexercise (n = 59)	Estimated meandifference	P value
**Depression**				
HAM-D_17_	11.3 (6.6)	10.5 (6.4)	−0.78 (−3.2 to 1.6)	0.52
HAM-D_6_	6.0 (4.0)	6.1 (3.4)	0.03 (−1.4 to 1.5)	0.97
HAM-A_14_	12.4 (7.5)	11.8 (7.4)	−0.54 (−3.3 to 2.2)	0.70
BDI	21.7 (13.9)	21.2 (15.1)	−0.52 (−5.6 to 4.5)	0.84
WHO-5	41.3 (24.0)	42.8 (25.5)	1.54 (−7.6 to 10.6)	0.74
**Cognitive function**				
Buschke’s SRT	12.4 (9.4)	13.4 (9.8)	1.0 (−2.1 to 4.1)	0.51
RCFT –3 min. recall	25.0 (6.5)	21.8 (6.8)	−3.2 (−5.5 to −0.9)	**0.007**
Digit span, forwards	7.0 (2.0)	6.9 (2.0)	−0.1 (−0.8 to 0.5)	0.68
Digit span, backwards	5.7 (2.0)	5.5 (1.9)	−0.2 (−0.8 to 4.2)	0.57
Subtracting Seriel Seven	7.4 (3.6)	7.9 (3.8)	0.5 (−0.8 to 1.9)	0.43
Stoop’s test, congruent	56.3 (14.8)	61.0 (15.9)	4.7 (−0.9 to 10.4)	0.10
Stroop’s test, incongruent	109.5 (32.0)	116.6 (34.8)	7.1 (−4.5 to 18.7)	0.23
Trail making A	27.4 (12.4)	28.6 (12.8)	1.2 (−3.2 to 5.7)	0.59
Trail making B	72.0 (31.0)	69.2 (32.1)	−2.8 (−13.3 to 7.6)	0.59
Digit symbol test	50.4 (8.5)	50.4 (8.9)	0.0 (−2.6 to 2.7)	0.98
Verbal fluency - animals	22.9 (5.0)	22.6 (5.2)	−0.3 (−1.9 to 1.3)	0.73
Verbal fluency – s words	13.6 (5.0)	14.1 (5.2)	0.5 (−1.1 to 2.2)	0.54
**Somatic**				
Weight, kg	76.2 (15.0)	78.3 (15.3)	2.1 (−0.8 to 5.1)	0.16
Body mass index, kg/m^2^	26.1 (4.9)	26.9 (5.0)	0.9 (−0.2 to 2.0)	0.12
Waist circumference, cm	87.0 (11.0)	89.2 (11.3)	2.2 (0.3 to 4.1)	**0.02**
VO_2 max_ ml/min/kg	29.3 (7.7)	24.9 (7.6)	−4.4 (−7.0 to −1.7)	**0.001**
Cholesterol, mmol/l	5.1 (1.1)	5.2 (1.1)	0.1 (−0.3 to 0.4)	0.66
Triglycerides, mmol/l	1.2 (0.7)	1.4 (0.7)	0.2 (−0.0 to 0.5)	0.06
HDL, mmol/l	1.4 (0.3)	1.4 (0.3)	−0.0 (−0.1 to 0.1)	0.95
Glucose, mmol/l	4.9 (0.7)	5.2 (0.7)	0.2 (0.0 to 0.5)	**0.04**
Insulin, pmol/l	71.1 (73.0)	79.2 (76.0)	−5.1 (−31 to 22)	0.71
QUICKI	0.76 (77.8)	0.74 (0.78)	−0.02 (−0.20 to 0.1)	0.74
HsCRP, mg/l	2.6 (3.5)	2.6 (3.8)	0.1 (−0.8 to 1.1)	0.78
Systolic BP, mmHg	118.6 (15.1)	116.8 (15.8)	−1.9 (−7.0 to 3.2)	0.47
Diastolic BP, mmHG	79.0 (9.2)	79.5 (9.7)	0.4 (−2.6 to 3.5)	0.78
**Work**				
Unemployed, n (%)	20 (35.7)	24 (40.7)	N/A	0.35
Sickness leave, n (%)	20 (35.7)	13 (22.0)	N/A	0.31
Job attendance, last 10 days, %	84.8 (28.5)	84.6 (26.0)	−0.24 (−15,7 to 15.2)	0.98

Post intervention. Data are presented with estimated mean (SD) unless stated otherwise. Estimation of missing data was performed using a maximum likelihood approach for continuous data and multiple imputations for dichotomous data. P values <0.05 are shown in bold. Abbreviations: HAM-D_17_/HAM-D_6_ – Hamilton depression scale with 17 or 6 Items; BDI – Beck’s Depression Inventory II; WHO-5 – World Health Organisation’s well being index; Buschke’s SRT – Buschke’s Selective Reminding Test; RCFT – Rey’s Complex Figure Test; VO_2 max_ – maximal oxygen uptake; HDL – high density lipoproteins; Quicki – Quantitative insulin sensitivity index; hsCRP – high sensitive C reactive Protein; BP – blood pressure.

The estimated number of patients in remission was 16/56 (28.6%) versus18/59 (30.5%) (chi-square = 0.05, df = 1; p = 0.82) in the aerobic and stretching exercise groups, respectively. There was no difference on other outcomes associated with depression, anxiety, or quality of life. In post-hoc analysis we found no moderating effect of sex (sex × time × group; F_1; 99_ = 0.108; p = 0.74), recurrent depression (recurrent depression × time × group; F_1; 100_ = 0.48; p = 0.49), and baseline HAM-D_17_ score ≥18 (high HAM-D_17_ × time × group; F_1; 101_ = 0.41; p = 0.52).

### Secondary Outcomes

Post intervention the maximal oxygen uptake (VO_2 max_) was 4.4 ml/kg/min higher (95% CI 1.7 to 7.0) in participants allocated to aerobic exercise compared to patients in the stretching control group. Participants allocated to the aerobic exercise group scored 3.2 points higher (95% CI 0.9 to 5.5) on the Rey Complex Figure test assessing the visuospatial memory. No other measure of memory or other assessed cognitive function differed post-intervention.

We found that the mean waist circumference was 2.2 cm lower (95% CI. 0.3 to 4.1) in participants allocated to the aerobic exercise compared with participants in the stretching control group. Participants in the aerobic group also had glucose levels 0.2 mmol/l lower (95% CI. 0.0 to 0.5) compared with participants in stretching control group.

We found no differences on job related outcomes in the aerobic versus the stretching exercise group.

### Adverse Events

Overall, four participants (8.5%) in the aerobic training group and eight (15.1%) participants in the stretching exercise group had started antidepressant medication treatment at follow-up (chi-square = 0.5; df. = 1; p = 0.33), and five patients in each group had a higher HAM-D_17_ rating at follow-up compared with the baseline assessment. As reported in table 3, there was one suicide attempt in the aerobic training group and none in the stretching exercise group during (p = 0.29) no significant differences between number of contacts to emergency services during the intervention. Adjusting the analysis of the primary outcome with the use of antidepressants did not influence the results (group × time × antidepressants; F_1; 110_ = 0.07; p = 0.79).

**Table 3:** Adverse events during a 3 months exercise intervention for patients with major depressive disorder.

	Aerobic exerciseN = 47	Stretching exerciseN = 53	P Value
Suicide attempt, n (%)	1 (2.1)	0 (0.0)	0.29
HAM-D_17_ rating worse at follow-up	5 (10.6)	5 (9.4)	0.93
Psychiatric emergency services	3 (6.4)	2 (3.8)	0.55
Admission to psychiatric department	2 (4.3)	2 (3.8)	0.90
Antidepressant medication	4 (8.5)	8 (15.1)	0.33

Data are presented as number of cases (% of total available at follow-up).

Abbreviation: HAM-D_17_ – Hamilton depression rating scale with 17 items.

### Completer Analysis

In the aerobic exercise group 55% of the participants attended more than 12 (33%) of the planned sessions. 39.3% of the participants attended more than 18 (50%) sessions and 19.6%more than 24 (66%) of the planned 36 sessions. In the stretching exercise group 47.5% of the participants attended more than 12 (33%) of the planned sessions, 35.6% of the participants attended more than 18 (50%) sessions and 15.3% more than 24 (66%) of the planned 36 sessions. Comparing the attendance in the two intervention groups, all p–values were above 0.40. A priori we defined completers as participants who attended 18 or more sessions. Restricting the analysis to these completers the estimated difference in HAM-D_17_ change was −0.18 (95% CI. −4.1 to 3.7; p = 0.93) in non-significant favor of stretching exercise. On all other outcomes the direction of estimated changes in the completer analysis were generally similar to the intention-to-treat analyses (table 2), but with wider confidence intervals and no significant effects.

## Discussion

The DEMO-II trial evaluated the efficacy of three months aerobic exercise intervention versus an attention control intervention in a group of mildly to moderately depressed adults referred from general practices. The trial data do not support any effect of aerobic exercise on depressive symptoms in this group. However, due to failure of enrolling the pre-defined number of participants, our results (non-significant as well as significant) should be interpreted with caution. We did find that participants allocated to aerobic exercise post-intervention had a significant higher maximal oxygen uptake (p = 0.001), visuospatial memory performance (p = 0.007), and a trend towards lower fasting glucose levels (p = 0.04) and waist circumference (p = 0.02) compared with participants in the control group. The intervention did not seem to have any effect on employment related outcomes.

The strengths of this trial are the randomization procedures, blinded outcome assessment, blinded statistical analysis, blinded preparation of the first manuscript draft, and the use of intention-to-treat analysis ensuring a low risk of bias. Moreover, the participants were all referred from a clinical setting to a pragmatic intervention supporting the generalisability and high external validity of this trial. However, the internal validity of the trial was compromised by a failure to include the pre-planned number of participants, which could potentially lead to random errors with under- or overestimation of effect sizes [33]. Furthermore, the low participant attendance could contribute to the lack of effect we found on depression outcomes. The failure to include the pre-planned number of participants was primarily due to a lower referral than anticipated. We used several activities to increase the number of referrals, and after additional 12 months we decided to stop inclusion of more patients due to financial constrains. We did use an attention control group as a comparator and not a no-treatment group. This has the benefit of isolating the potential biological effect of aerobic exercise on depressive symptoms [34], but also introduces a risk of contamination in the control group. As an argument against the latter possibility, the maximal oxygen uptake did not improve in the control group compared with baseline data, which suggests that this group was not ‘contaminated’ by increased fitness levels. This provides us with an increased internal validity in terms of understanding the potential antidepressant effect of aerobic exercise. On the other hand, it could be argued that with the observed remission rates stretching exercise potentially offers a true antidepressant effect. However, to our knowledge no proper pre-clinical or clinical studies have yet been conducted that reasonably could support such a notion. To exclude this possibility further randomized clinical trials could be conducted.

The neutral findings of the current trial in regard to our primary outcome, the mean difference in HAM-D_17_, is in accordance with other similar studies [35]–[37] as well as our previous trial [13] Two recent meta-analyses [8], [38] both estimated a small to moderate effect of exercise in depressed populations. When only including trials with low risk of bias in the meta-analysis (i.e., adequate randomization procedure, intention-to-treat analysis, and blinded outcome assessment), however, the effect size in both meta-analyses showed small and non-significant antidepressant effects of exercise. A smaller trial found highly significant dose-response effects of exercise aerobic exercise compared with a control group on both HAM-D_17_ mean difference and number of patients in remission [39]. In that particular trial the attendance rates were >70%, which is in accordance with other similar trials [35], compared to an average attendance of only 36% in the current trial, corresponding to a mean attendance of one session per week. We have scrutinized other trial reports but not been able to identify differences in handling of participants [35], [39]. Except that the majority of other trials recruit participants directly to the research institute and not from a clinical setting as in the current, which could potentially explain different attendance behavior. On the other hand, the increase in maximal oxygen uptake from baseline was 13.1% in the current trial compared to 7.1% [37] and 8.3% [35] in other trials suggesting a robust physiological intervention effect despite the low average participation. A descriptive study from UK found that only 22% of participants referred on basis of mental health issues (i.e., anxiety or depression) to exercise schemes participated in 80% of weekly or biweekly sessions in eight to twelve weeks programs [40]. In comparison, 50% of the participants in the DEMO-II trial attended weekly and 14% attended biweekly, which suggests that the attendance rate observed in the current study reflects a ‘real-life setting’ enhancing the external validity of this trial. A similar pragmatic exercise intervention for patients with long-term neurological conditions also had an average attendance of one session per week [41]. However, the limited attendance to our study does compromise the validity to this study in terms of answering the question: Is exercise a use-full antidepressant? The study by Trivedi et al. [37] suggests that the effect of exercise on depressive symptoms could be modulated by family history of mental illness and sex. We did not find that potential modulators such as sex, recurrent depression, or a high baseline HAM-D_17_ (HAMD ≥18) had any influence on the results. Unfortunately, we did not collect data on family history of mental illness.

Spatial memory is associated with hippocampal function [42] and increases in hippocampal volume have been found in response to exercise in healthy individuals [43], [44] patients with schizophrenia [45] and older persons [46]. Potentially the increase in spatial memory we observed could be explained by exercise related changes in hippocampal function as observed in a trial with older persons [46]. In concurrence with our previous trial [13], we did not find any effect of exercise on any other cognitive function, which could suggest that the positive finding on spatial memory could be a spurious finding. Only one other group have reported on the effect of exercise on cognitive function in two different trials [14], [47]. In one trial of older persons with depression the group found exercise to have positive effects on pooled estimates of memory and executive function, and in the second which [14] In the second trial of middle-aged patients with depression [47] they found no effect of exercise on cognition, which is in line with the current trial. The trial with positive results included old persons, which is a group that potentially has cognitive benefits from aerobic exercise in selected cognitive domains such as motor function, cognitive speed, and auditory and visual attention. Also, adult outpatients with mild to moderate depression do not necessarily exhibit cognitive impairment and the lack of effect could be ceiling effect.

To our knowledge this is the first randomized trial to show a tendency for improved metabolic fitness in patients with depression (i.e., tendency for lower waist circumference, lower fasting plasma glucose and insulin) as a result of aerobic exercise. Looking at evidence from high risk populations these are not surprising findings [48]. These findings have two valuable consequences in terms of understanding the current results. Despite the physiological changes, including increased maximal oxygen uptake, we did not find an antidepressive effect of exercise. It is possible, in this group of hard-to-motivate patients, to obtain relevant changes in variables associated with the metabolic syndrome.

Peripheral inflammatory responses can affect the immune system by direct or indirect pathways, possibly mediated by cytokines and other immune components [49]. In combination with the observed link between increased C reactive protein and subsequent depression [50], this has given rise to a theory implying a possible role for cytokines and other immune components in depression, either as a cause or as an epiphenomena [51].

However, we were not able to demonstrate an anti-inflammatory effect of exercise which have been found in other trials [52] of non-depressed individuals. This could potentially explain our findings in terms of no antidepressant effect of exercise in the current study.

We found no effect on any employment related outcome in the present trial. In the DEMO-I trial [13] this effect was primarily observed after 12 months from baseline and in participants allocated to resistance training.

The DEMO-II trial does not support referring patients from general practices to a 3 months exercise program to obtain antidepressant effects. However, the trial had to be prematurely terminated and we cannot exclude a type II error. Also, the current trial does suggest that an exercise program for patients with depression might offer positive short-term effects on maximal oxygen uptake, waist circumference, and fasting plasma glucose levels. Future research on the effect of exercise and depression should be designed to address the effect of exercise in sub-groups (i.e., patients with or without genetic disposition for depression). Secondly, research should focus on creating exercise programs, which address motivational issues for increased adherence in this patient group and variables associated with the metabolic syndrome.

## Supporting Information

Checklist S1 **CONSORT Checklist.**
(DOC)

Protocol S1 **Trial Protocol.**
(DOC)
