# Supplementary material for: DEMO-II Trial. Aerobic Exercise versus Stretching Exercise in Patients with Major Depression—A Randomised Clinical Trial
Source: PLoS One. 2012 Oct 31;7(10):e48316. doi: 10.1371/journal.pone.0048316 (PMC3485141; doi:10.1371/journal.pone.0048316)
Supplement: Protocol S1 — Trial Protocol. (DOC) [file pone.0048316.s002.doc]

DEMO II: A randomized, parallel-group, observer-blinded clinical trial of aerobic exercise versus stretching exercise for patients with light to moderate depression

Substudy: The effect of exercise on hippocampal volume

# Purpose

# The primary objective for the DEMOII superiority trial is to assess the beneficial and harmful effect of aerobic exercise versus stretching exercise in patients diagnosed with moderate depression according to DSM-IV (Diagnostic Interview Schedule – IV).

# Background

Projecting the current development in disease patterns unipolar depression is expected to be the second highest cause of global disease burden in 2030^1^. With remission rates of approximately 50% using medical antidepressant therapy^2, 3^, the search for alternative or augmentation therapy is a key issue in depressive research. Furthermore, Major depression is associated with increased mortality from somatic disorders such as cardiovascular disease, stroke, and endocrinological diseases^4^.

In 2001 a meta-analysis concluded that the effect of exercise training could not be determined due to lack of good quality research^5^. The authors found that the majority of trials were without blinded outcome assessment, lacked intention-to-treat analysis, and most had short follow-up. In this context, one of the authors in 2004 launched a pragmatic randomized controlled trial investigating the effect of exercise training on depressive symptoms – the DEMO1 trial. The trial included 165 participants who were randomized to either aerobic, non-aerobic, or relaxation training in a four-month training programme. These results did not support any biological effect of exercise on major depression. However, the majority (115/165) of patients had received antidepressant medication for more than six weeks at baseline thus the lack of a positive result could partly be explained by a treatment resistant group. A subgroup analysis of patients not medicated at baseline estimated the effect between non-aerobic and relaxation training to -1.7 (95% CI: -6.0 to 2.5) and between aerobic and relaxation training to 0.3 (95% CI: -3.9 to 4.6). The low number of patients in this important sub-group, means that any conclusion on non-medicated patients is underpowered.

On this background we have designed this randomized trial comparing aerobic exercise and no treatment in light to moderate depression. This trial will employ adequate methods of reducing bias such as centralized randomization, blinded outcome assessment, and analysis according to the intent-to-treat principle.

# Methods

Design

The trial is designed as a two-armed, parallel-group, observer-blinded randomized clinical superiority trial. The patients are randomized to either aerobic exercise or a stretching exercise program. The stretching exercise program will function as a control group with minimal muscle contractions, and is established in order to eliminate the potential positive effect of being in a group on a regular basis.

# Subjects

Patients will be referred from general practitioners and private practicing psychiatrists to the first interview, which determines if the patient fulfils the inclusion criteria and none of the exclusion criteria.

The inclusion criteria is age between 18 and 60, living in the vicinity of Copenhagen, moderate depression according to DSM-IV, and have to be able to read and understand informed consent. Exclusion criteria is current or recreational drug abuse, having used antidepressant medication within the last 2 months, contradictions to physical exercise, regular recreational exercise > 1 hour/week, suicidal behaviour (HAM-D_17_ item 3 >2), medication for diabetes, hypertension, or any kidney disease.

# Clinical assessment

A research assistant will conduct the interview of the participants. The diagnosis of depression is established using the Mini International Neuropsychiatric Interview^6^, and the severity of depression by HAM-D_17_^7^. The self-administered depression rating scale Becks Depression Inventory^8^ will be used in addition.

# Physical exercise testing

The patients’ maximal oxygen uptake (VO_2max_) will be measured using a bicycle cardiopulmonary exercise test. The test is based on L. B. Andersen’s cycle exercise protocol^9^. The patients is instructed to fast from midnight prior to exercise testing, meet between 8:30 and 10:00 a.m., and not to engage in any strenuous physical activity prior to testing. In the initial 5 min of the test the aim is a workload inducing a pulse of 120 beats per minute, and then the workload will increase with 25 watt/2min. The speed will be 65 rounds pr. minute. An indwelling venous catheter is inserted in the antecubital vein and blood samples are collected after 5 min. rest in a sitting position. Blood samples will again be collected at VO_2max_ and 15, 30 and 60 minutes after the exercise test. All blood samples that are not immediately analyzed are centrifuged and the plasma (serum for IGF-1) stored at –80 °C until analyzed. Weight, height, blood pressure and waist circumference will be assessed by standardized procedures.

# Randomization

If the inclusion criteria are fulfilled and none of the exclusion criteria is fulfilled the patients are randomized prior to the first training session. Randomization will be centralized and carried out by the Copenhagen Trial Unit. The block size will be unknown to investigators of the trial. The physiotherapist in the trial will by phone contact Copenhagen Trial Unit for participant placement allocation.

Randomisation of the participants will be stratified according to two criteria: 1) the Hamilton score (>19), and 2) high systolic (>150 mmHg) or diastolic (>95 mmHg) blood pressure.

# Interventions

Both intervention groups and healthy volunteers will meet three times pr. week for three months.

The program in the aerobic training group is designed to increase fitness as measured by maximal oxygen uptake (VO_2max_) in a maximal cycle ergometer test. After initial 10 min. of general low-intensity warm-up the participants will do 30 minutes of aerobic exercise on a stationary cycle ergometer (Monark). The first 4 weeks they will work at an intensity level corresponding to 65% of their VO_2max_, the next 4 weeks at 70% and in the last four weeks at 75 to 80% of their VO_2max_. The participants will carry puls monitor (Polar m-31) during exercise sessions to guide and document intensity levels. Then 5 minutes low-intensity cool down period.

Stretching exercise. Initial 10 min. of low-intensity warm-up, followed by 30 minutes program of stretching and very low intensity exercises.

# Outcomes

### Primary outcome

The HAM-D_17_^7^ is chosen as the primary outcome due to its widespread international use in psychiatric hospitals and among general practitioners. The assessor will be blinded to intervention group. At the follow-up interviews, the patients are instructed not to reveal their group assignment. At the follow-up the interviewer will ‘guess’ which group the patients are allocated to, making it possible to reveal if the ‘blinding’ is successful. Intraclass correlation between raters using analysis of variance will be performed^10^. The self-administered Becks depression inventory^8^ will also be applied.

Hypothesis:

1. The reduction in HAM-D_17_ score post intervention will be higher in the aerobic training group compared to stretching exercises.

Sample size calculation

We look for a 3 point difference on the HAM-D_17_. Based on our previous trial we estimate SD to 6 on this parameter. With an alpha of 0.05 and beta at 0.9 we need 85 participants in each arm. Based on our experience in this we expect a drop-out of approximately 20% post-intervention. We therefore aim to include a total of 212 participants.

### Secondary outcome, cerebral blood volume in dentate gyrus

In a study by Pereira et al.^11^ they showed a positive correlation between BrdU labeled cells and changes in CBV measured by magnetic resonance in the mouse dentate gyrus (hippocampus) after an exercise intervention. CBV in dentate gyrus have previously been correlated to cognitive function in monkeys^12^. The same MRI technique in humans and found that exercise increased CBV in the dentate gyrus and that it was correlated with cardiopulmonary function and cognitive skills, supporting pre-clinical studies of exercising rats leading to increased neurogenesis^13^ in hippocampus.

The difference in pre- and post contrast images will be used to assess CBV. Due to possible differences in global blood flow^14^ and level of contrast administration, the differences in signal intensity will be normalized using the maximum signal value of the sagittal sinus^15^. The mean relative CBV (rCBV) pre-post intervention will be used to compare groups. Coronal T1-weighted images (repetition time, 20ms; thickness 4mm) oriented perpendicular to the hippocampal long-axis before and 4 minutes after i.v. administration of the contrast gadolinium (0.1 mmol/kg). We will use a 3-Tesla scanner with 3-D gradient echo sequence. Prior to testing all patients will be screened for kidney function with s-creatinine.

Hypothesis

1. Post-intervention rCBV will be higher in the aerobic group compared to the control group.

Sample size estimation

Based on the article by Pereira et al.^11^ we can expect the rCBV to increase 0.5 with a SD of 0.25. We look for a post intervention difference of 0.25 in rCBV. With alpha to 0.05 and beta to 90%, and an expected dropout of approx. 20% we need 26 participants in each group. The first 26 recruited patients in each group will be recruited for this.

### Tertiery outcomes

In addition to fCBV we will measure any changes in hippocampal (dentate gyrus) volume using two pulse sequences: One T1-weighted 3D MPR, with a 1*1*1 mm resolution, and one T2-weighted SPACE sequence with a 1*1*1 mm resolution.

Risk factors associated with development of cardiovascular disease will be measure prior and after the intervention (BMI, waist/hip ratio, cholesterol, bloodpressure, c reactive protein, interleukin 6) by standardized procedures.

Cognitive abilities will be tested by standardized procedures prior and after the intervention. Testing of cognitive skills will include areas of verbal intelligence, attention, visuomotor speed, language and memory.

# Statistics

The statistical analysis will be based on the intention-to-treat principle including all patients that have been randomized. We will conduct our analysis using a likelihood-based, mixed model, repeated measurement method with unstructured variance. Per-protocol analysis will be undertaken including participants who have been at more than 50% of the sessions.

# Organization and funding

The intervention and screening of patients will take place at Bispebjerg Hospital in Copenhagen. The hospital provides training and office facilities.

Training and exercise testing will be done by a physiotherapist. The screening and psychometric evaluation will be done by a nurse and a research assistant.

MRI will be done in cooperation with Rigshospitalet in Copenhagen.

The author group consist of researchers with expertise in exercise, depression, clinical trials, and MRI. Furthermore, part of the author group have just ended collecting data from a much similar trial, thus the expertise for the management of this trial is very much present.

# Publications

The results will be published in international peer-reviewed journals, and offered to Danish medias.

# Ethical issues

Only limited harm is expected to the patients in terms of muscle tenderness, venous puncture and side effects of non-ionic contrast agent gadolinium such as nausea and dizziness. In case the depression severely worsens, the patient will be referred to an independent psychiatrist for evaluation. The trial protocol is approved by the local ethical committee (H-A-2008-046), and the Danish Data Protection agency (j.nr. 2008-41-2354), and registered at ClinicalTrials.gov (NCT 00695552). The patients will be requested to give written informed consent after having received written and oral information on the trial. The trial will be conducted in accordance with the Helsinki Deklaration.
